# High-Coverage Reconstruction of XCO_2_ Using Multisource Satellite Remote Sensing Data in Beijing–Tianjin–Hebei Region

**DOI:** 10.3390/ijerph191710853

**Published:** 2022-08-31

**Authors:** Wei Wang, Junchen He, Huihui Feng, Zhili Jin

**Affiliations:** School of Geosciences and Info-Physics, Central South University, Changsha 410017, China

**Keywords:** satellite, remote sensing, CO_2_, mapping, random forest

## Abstract

The extreme climate caused by global warming has had a great impact on the earth’s ecology. As the main greenhouse gas, atmospheric CO_2_ concentration change and its spatial distribution are among the main uncertain factors in climate change assessment. Remote sensing satellites can obtain changes in CO_2_ concentration in the global atmosphere. However, some problems (e.g., low time resolution and incomplete coverage) caused by the satellite observation mode and clouds/aerosols still exist. By analyzing sources of atmospheric CO_2_ and various factors affecting the spatial distribution of CO_2_, this study used multisource satellite-based data and a random forest model to reconstruct the daily CO_2_ column concentration (XCO_2_) with full spatial coverage in the Beijing–Tianjin–Hebei region. Based on a matched data set from 1 January 2015, to 31 December 2019, the performance of the model is demonstrated by the determination coefficient (R^2^) = 0.96, root mean square error (RMSE) = 1.09 ppm, and mean absolute error (MAE) = 0.56 ppm. Meanwhile, the tenfold cross-validation (10-CV) results based on samples show R^2^ = 0.91, RMSE = 1.68 ppm, and MAE = 0.88 ppm, and the 10-CV results based on spatial location show R^2^ = 0.91, RMSE = 1.68 ppm, and MAE = 0.88 ppm. Finally, the spatially seamless mapping of daily XCO_2_ concentrations from 2015 to 2019 in the Beijing–Tianjin–Hebei region was conducted using the established model. The study of the spatial distribution of XCO_2_ concentration in the Beijing–Tianjin–Hebei region shows its spatial differentiation and seasonal variation characteristics. Moreover, daily XCO_2_ map has the potential to monitor regional carbon emissions and evaluate emission reduction.

## 1. Introduction

The global atmospheric CO_2_ concentration has increased dramatically since the industrial revolution. From ground observation, the atmospheric CO_2_ concentration has increased from 280 ppm at the beginning of the industrial revolution to 413.2 ppm in 2020 [1] and is also rising at a rate of nearly 2 ppm every year [2]. With the increase in atmospheric CO_2_ concentration, the global greenhouse effect is also increasing [3], and extreme weather and natural disasters are frequent [4]. Accurately estimating and effectively responding to the change in atmospheric CO_2_ concentration are major scientific issues to achieve the earth’s sustainable development [5]. Atmospheric CO_2_ column concentration (XCO_2_) is often used to represent atmospheric CO_2_ concentration [6]. Atmospheric XCO_2_ concentration can be measured in two ways: (1) Observing CO_2_ concentration based on ground stations: The total carbon column observing network (TCCON) established by the American Center for Atmospheric Research in 2004 can provide long-time and high-precision XCO_2_ concentration and effectively reveal the spatiotemporal variation trend of XCO_2_ concentration [7]. However, accurately representing the spatial distribution and temporal changes in XCO_2_ concentration by a few TCCON stations is difficult [8]. (2) Observing CO_2_ concentration based on remote sensing satellites: XCO_2_ concentration with high spatial–temporal resolution can be provided by remote sensing satellites [9], which have large-scale and long-time-series advantages. Currently, widely used CO_2_-monitoring satellites include GOSAT [10], OCO-2 [11], TanSat [12], and so on.

Although a remote sensing satellite has many advantages in monitoring XCO_2_ concentration, it inevitably has some problems. (1) The monitored scope is limited by the satellite observation mode [13]. (2) Satellites can be easily influenced by cloud cover and aerosols [14]. For example, the valid observation of the OCO-2 satellite only account for about 10% of all observation after quality control [15]. Currently, the coverage of atmospheric XCO_2_ monitored by satellites is low. This low coverage of XCO_2_ concentration has a negative influence on accurately estimating the carbon source and sink is difficult [16].

Researchers have developed various methods to reconstruct the high coverage of XCO_2_ data [17]. A high-accuracy surface modeling method was used to reconstruct the high coverage of OCO-2 XCO_2_ data [18]. Monthly XCO_2_ concentration can be obtained using the middle and low latitudes of the world. Additionally, the Goddard Earth Observing System Chemistry model has been used to obtain XCO_2_ concentration with continuous space–time coverage based on the atmospheric driving method [19,20]. However, the spatial resolution of the XCO_2_ concentration data obtained by the above method is generally above 0.5°, which cannot support the detailed study of regional carbon sources and sinks [21].

Machine learning algorithms can effectively deal with nonlinear complex system problems [22,23] and have been widely used in atmospheric XCO_2_ concentration estimation models. For example, the artificial neural network (ANN) method and variables (e.g., longitude and latitude, sea temperature, salinity level, and chlorophyll-a concentration) were used to model the XCO_2_ concentration over the ocean [24]. Siabi and Falahatkar modeled the 5 km seamless XCO_2_ concentration over Iran using the ANN method [25], OCO-2 XCO_2_, and eight environmental variables, including the normalized difference vegetation index (NDVI), net primary productivity (NPP), leaf area index, land surface temperature, wind direction, wind speed, air temperature, and land cover type.

Tarko and Usatyuk [26] showed that the temporal and spatial distributions of atmospheric CO_2_ concentration are affected by multiple factors, among which atmospheric meteorological conditions, vegetation carbon sink absorption, and carbon emissions from human activities are the most significant factors. Focusing on the aforementioned three types of variables is necessary to obtain more accurate XCO_2_ concentration [27].

Thus, this study aimed to obtain a high-coverage and high-spatial–temporal resolution atmospheric XCO_2_ concentration based on a machine learning model by integrating multisource remote sensing satellite data, considering meteorological factors, anthropogenic emissions, natural carbon sinks, and so on. Then, spatial–temporal changes in regional XCO_2_ concentration were analyzed. Simultaneously, the geographical locations of regional carbon sources and sinks are explored.

## 2. Data and Methods

### 2.1. Study Area and Data

#### 2.1.1. Study Area

**Figure 1:**
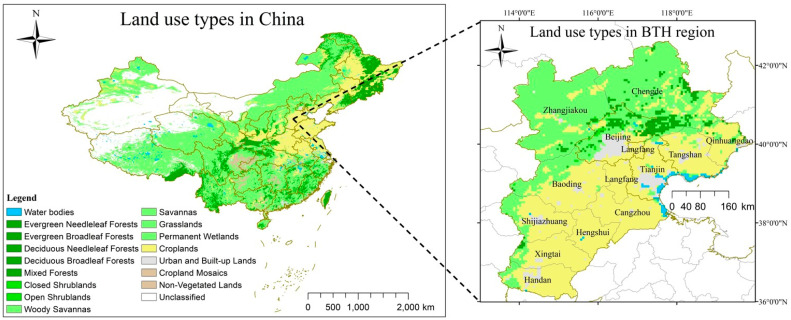
Land use types in the Beijing–Tianjin–Hebei (BTH) region in China.

The study area was the Beijing–Tianjin–Hebei region in the North China Plain. The Beijing–Tianjin–Hebei region is centered in Beijing, the capital of China, including Tianjin, Shijiazhuang, Tangshan, Handan, Baoding, Cangzhou, Xingtai, Langfang, Chengde, Zhangjiakou, Hengshui, and Qinhuangdao, with a total area of 216,000 km^2^. The land use type map of the study area in 2014 is shown in Figure 1. The land use data are from the MODIS Land Cover (MCD12Q1) Product, which can be downloaded from https://lpdaac.usgs.gov/products/mcd12q1v006, accessed on 1 September 2021.

Li et al. [28] pointed out that population size has a great impact on carbon emissions. Beijing and Tianjin are the second and third largest cities in China, respectively, with developed industries and a large population [29]. The Beijing–Tianjin–Hebei region has become a typical high-carbon-emission region in China. Thus, reconstructing high-coverage XCO_2_ map in the Beijing–Tianjin–Hebei region is necessary.

#### 2.1.2. Data

1. OCO-2 XCO_2_

**Figure 2:**
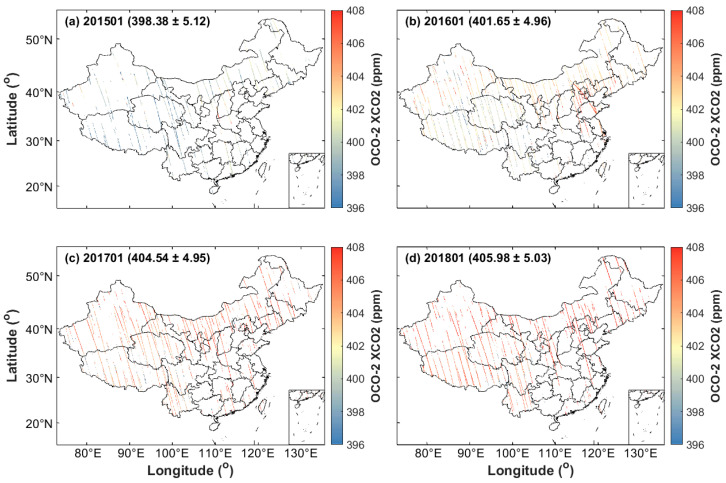
Average monthly data of OCO-2 XCO L2 Lite_FP in China: (**a**) January 2015, (**b**) January 2016, (**c**) January 2017, and (**d**) January 2018.

Following the failure to launch the carbon olfactory satellite (OCO) in 2009, the National Aeronautics and Space Administration launched the OCO-2 satellite in 2014 to monitor the change in atmospheric XCO_2_ concentration [30]. The level 2 product published on the official website (https://search.earthdata.nasa.gov, accessed on 10 February 2021) was used in this study. The spatial resolution and the measured period of this product are 1.29 km × 2.25 km and 16 days, respectively [13]. The OCO-2 level 2 product includes three XCO_2_ products, namely, V7, V7r, and Lite_FP file products. For data applications, Lite_FP was selected in this study because it usually has the most effective data volume and relatively stable spatial coverage. Liang et al. [31] showed that OCO-2 XCO_2_ has a random error of ~1.8 ppm compared with ground-based TCCON data, which was sufficient to improve the estimation of the carbon source and carbon sink. Obviously measured gaps in XCO_2_ retrievals due to the influence of the observation orbit, cloud coverage, and aerosols (Figure 2).

2. VIIRS S-NPP

The level of regional economic development is closely related to the population size and the industrial development level, which are closely related to the magnitude of anthropogenic carbon emissions [32]. The mean value of lighting data can effectively reflect the overall economic development level of the region and then effectively reflect the magnitude of anthropogenic carbon emissions [33].

The visible infrared imaging radiometer (VIIRS) night-light data used in this study is an extension of the MODIS series and is carried on the S-NPP satellite [34]. Global daily measurement of night-visible and near-infrared light can be provided by VIIRS, with spatial and time resolutions of 500 m and 1 day, respectively. Level-3 data were used in this study. This level of data has been geometrically and radiometrically corrected and can be downloaded from https://search.earthdata.nasa.gov, accessed on 21 October 2020.

Atmospheric CO_2_ is distributed in the form of aggregation and fog. The difference of XCO_2_ concentration within a certain range is small, while the night-light values of different grid points are very different. The point-to-point matching mode cannot effectively correspond to the XCO_2_ concentration. Therefore, the mean night-light value was adopted to represent the overall emissions in a region.

Firstly, the four-scene noctilucent data were spliced to obtain the complete lighting data in the Beijing–Tianjin–Hebei region. Then, the lighting map was resampled to 0.05° × 0.05°. The sum of lighting value in each city was counted and then divided by the region area of each city to obtain the average value. The formula is as follows:

(1) $${\mathrm{DN}}_{\mathrm{mean}}=\frac{{\mathrm{DN}}_{\mathrm{all}}}{{\mathrm{Area}}_{\mathrm{city}}}$$

where ${\mathrm{DN}}_{\mathrm{all}}$ is the sum of lighting value in a city; ${\mathrm{Area}}_{\mathrm{city}}$ is the area of the city, counted by the number of pixels; and ${\mathrm{DN}}_{\mathrm{mean}}$ represents the mean value of the city’s lighting data.

**Figure 3:**
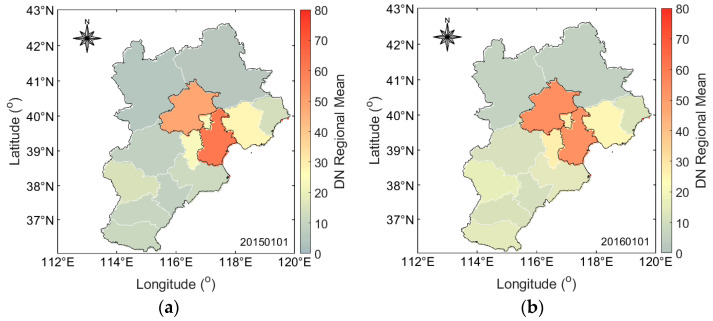
Regional mean value results of VIIRS S-NPP luminous data. The left and right figures show the regional mean value results of luminous data on (**a**) 1 January 2015 and (**b**) 1 January 2016, respectively.

The same processing was performed on the light data for each day from 1 January 2015 to 31 December 2019. Examples of regional mean light values are shown in Figure 3.

3. Natural carbon sink

As an important part of the carbon sink, the growth status and spatial coverage of surface vegetation have a very significant impact on atmospheric CO_2_ concentration [35,36]. In this study, the NDVI was used to characteristic the vegetation growth status and vegetation coverage. The calculation formula is shown in Equation (2). The NDVI data used in this study are from Terra’s MODIS sensor, with spatial and time resolutions of 500 m × 500 m and 16 days, respectively, downloaded from https://search.earthdata.nasa.gov, accessed on 10 October 2021.

(2) $$\mathrm{NDVI}=\frac{\mathrm{NIR}-\mathrm{Red}}{\mathrm{NIR}+\mathrm{Red}}$$

where, NIR and Red are the near-infrared band and red band surface reflectance, respectively.

4. Meteorological factors

In this study, the impact of meteorological parameters on atmospheric CO_2_ concentration was also considered in addition to selecting the influencing factors of carbon sources and sinks of anthropogenic emissions and natural vegetation [24,25,37]. As one of the atmospheric chemical components, the temporal and spatial variations in CO_2_ concentration are greatly affected by meteorological factors. The meteorological factors affecting the concentration of atmospheric chemical components mainly include wind speed, temperature, and atmospheric stability. Such as, wind can dilute the atmospheric molecules. The temperature can reflect the stability of the atmosphere. In winter, the temperature is low, and the atmospheric structure is relatively stable, which is not conducive to the vertical diffusion of pollutants.

Five meteorological factors, including temperature (TEMP), relative humidity (RELH), pressure (PRES), wind speed (WS), and boundary layer height (BLH), were selected. Meteorological data from the European Meteorological Center reanalysis data set (ERA5) were used in this study. These are the fifth-generation ECMWF global climate data for atmospheric reanalysis. The spatial resolution of ERA5 data used in this study is 0.25° × 0.25° with a time resolution of 1 h, which can be downloaded from the ECMWF official website (https://cds.climate.copernicus.eu, accessed on 3 June 2021). All meteorological data were resampled to a resolution of 0.05° to fit the OCO-2 XCO_2_ data by a bilinear interpolation method in this study, and the meteorological data at 13:00 local time were selected to match the XCO_2_ data.

Table 1 shows the data sets used in this study.

5. Time series variables

Relevant studies have shown that the atmospheric CO_2_ concentration has obvious seasonal variation characteristics. Keeling et al. [38] put forward the classical formula for the variation in atmospheric CO_2_ concentration over time:

(3) $$y=A_{1}\sin2\pi t+A_{2}\cos2\pi t+A_{3}\sin4\pi t+A_{4}\cos4\pi t+A_{5}+A_{6}t$$

In the above formula, $A_{1}$ − $A_{4}$ determines the periodic change law of atmospheric CO_2_ concentration with seasons, $A_{5}$ determines the background atmospheric CO_2_ concentration, and $A_{6}$ represents the interannual linear increment. t represents the time from the start date in years, and y represents the XCO_2_ concentration in ppm.

In this study, the seasonal variation characteristics of atmospheric CO_2_ concentration were also considered, and time series variables were added to the model to improve performance.

### 2.2. Methodology

#### 2.2.1. Methodological Process

**Figure 4:**
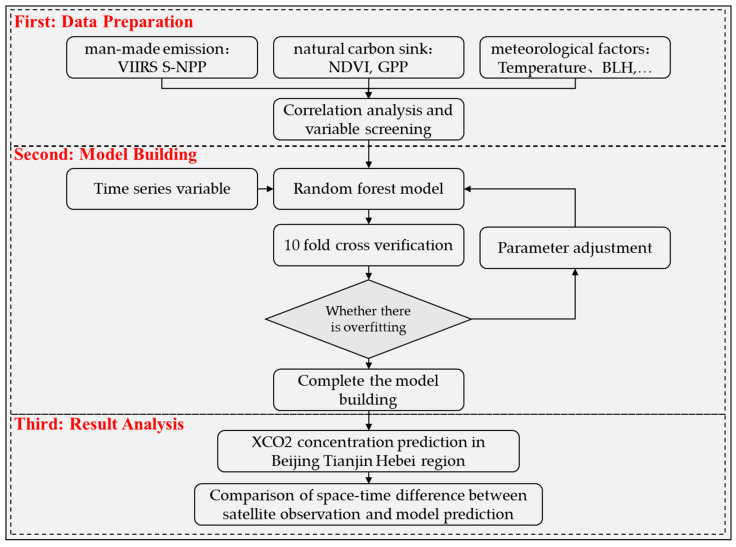
Flow chart of this study.

The flow chart of this study is shown in Figure 4, which mainly consists of three parts.

The first part was mainly to obtain the data and screen the model variables. By analyzing influence factors of atmospheric CO_2_ and the correlation between the variables and XCO_2_ concentration, the appropriate variables were selected to build the model.

The second part was mainly to build the model and verify the accuracy, including select the appropriate algorithm to build the model, and use statistical indicators to evaluate the model’s results. Finally, cross-validation was used to check whether the model overfitting or not.

The third part was mainly to compare and analyze the spatio-temporal differences between the XCO_2_ data set simulated by the model and the XCO_2_ data set monitored by the satellite.

#### 2.2.2. Random Forest Model

The atmospheric system is a complex system with uncertainty. The number of atmospheric molecules (e.g., CO_2_) is influenced by different atmospheric conditions. For example, CO_2_ near the ground can be rapidly transported to the upper air and surrounding areas in summer due to intense atmospheric convection. In addition, some gases containing the element C, such as CO and CH_4_, will be converted into CO_2_ under the action of atmospheric chemistry for a long time. Therefore, certain limitations were observed in modeling and estimating CO_2_ concentration using the mechanism model. A neural network algorithm has a strong nonlinear and self-learning ability. However, it has some problems (e.g., slow convergence, serious overfitting, and so on) for the estimation of high-dimensional features and needs to continuously optimize the model parameters to achieve optimal results [39].

The random forest model selected in this study, which was first proposed by Cutler et al. [40]. It is an integrated algorithm, including multiple decision trees. The stochastic forest model has the following advantages:

1. The model has few adjustment parameters and does not require too much time.
2. The random selection of sample sets and split attributes can effectively reduce the overfitting of the model.

Through the continuous implementation and verification of the fitting results of the model, the random forest model established in this study mainly adjusts two important parameters: the maximum depth of the decision tree and the minimum number of samples of leaf nodes. The deeper the decision tree is, the longer time the model takes, but the model performance may be improved to some extent. In this research model, the maximum depth of the decision tree was set to 30. The larger the minimum number of leaf nodes, the smaller the branches of the decision tree, and it has a certain ability to resist overfitting. However, as the minimum number of leaf nodes increases to a certain extent, the accuracy of the decision tree will be difficult to guarantee. Through continuous experiments, the minimum number of samples of leaf nodes was set to 3 in the model.

#### 2.2.3. Data Resampling and Matching Method

In the process of building the model, bilinear interpolation was used to uniformly sample with a spatial resolution of 0.05°. The matched data include XCO_2_ concentration, VIIRS S-NPP, NDVI, temperature, relative humidity, atmospheric pressure, wind speed, and boundary layer height. By matching the data from 1 January 2015 to 31 December 2019, 62,964 samples were obtained. Subsequently, the matched samples were used for model training and verification.

#### 2.2.4. Model Validation Method

In this study, in addition to the direct fitting results of model, the model was also verified by tenfold cross-validation (10-CV), which can avoid the potential overfitting in the model. After randomly dividing 62,964 pieces of data into 10 subparts, 9 of them were used for training, and 1 was used for estimation. The estimated results were compared with the measurements, the process was repeated ten times until each piece of data was estimated, and finally, the estimated values of all data were obtained.

The determination coefficient (R^2^), root mean square error (RMSE), mean absolute error (MAE), and other statistical indicators were used to evaluate the accuracy of the model. The formulas of R^2^, RMSE, and ME are as follows:

(4) $$R^{2}={\left(\frac{\sum_{i=1}^{n}(x_{i}-\bar{x})(y_{i}-\bar{y})}{\sqrt{\sum_{i=1}^{n}{\left(x_{i}-\bar{x}\right)}^{2}\cdot \sum_{i=1}^{n}{\left(y_{i}-\bar{y}\right)}^{2}}}\right)}^{2}$$

where x and y represent the satellite-based and model estimated XCO_2_, respectively, $\overset{¯}{x}$ represents the mean XCO_2_ value observed by the satellite, $\overset{¯}{y}$ represents the mean XCO_2_ value estimated by the model, and n represents the number of samples.

(5) $$\mathrm{RMSE}=\sqrt{\frac{\sum_{i=1}^{n}{\left(X_{i}-\hat{X}\right)}^{2}}{n-1}}$$

where $X_{i}$ represents model fitting results, $\hat{X}$ represents the mean value of model fitting, and n represents the total number of samples.

(6) $$\mathrm{MAE}=\frac{1}{n}\sum_{i=1}^{n}|\hat{Y}-Y_{i}|$$

where $Y_{i}$ represents model fitting results, $\hat{Y}$ represents the mean value of the model fitting results, and n represents the total number of samples.

## 3. Results

### 3.1. Descriptive Statistics

**Figure 5:**
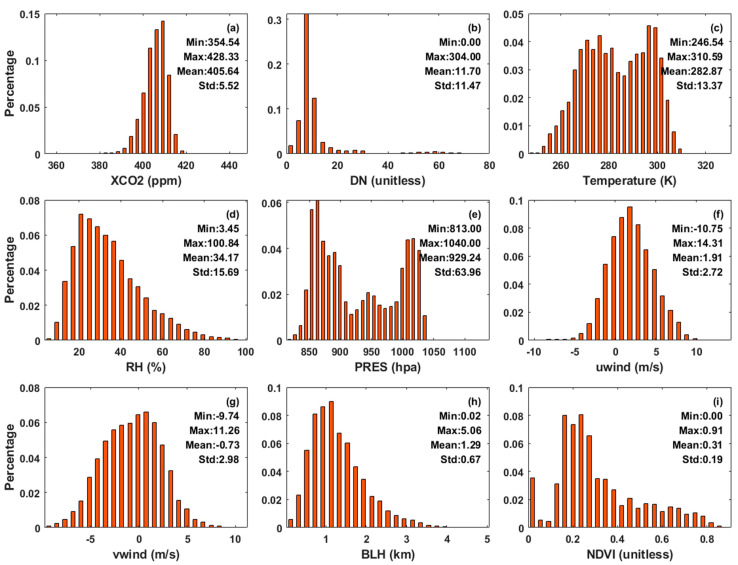
The frequency histogram of parameters in XCO_2_ concentration modeling (n = 62,964). (**a**–**i**) represent CO_2_ column concentration, digital number, temperature, relative humidity, pressure, vertical wind speed, horizontal wind speed, boundary layer height, and normalized vegetation index, respectively.

Before modeling, the above-mentioned various types of data were matched one by one according to longitude, dimension, and time, and a total of 69,512 pieces of data were matched. Statistical analysis of the 62,964 matched data was performed y to avoid problems in the data preprocessing process. The frequency histogram of each parameter is shown in Figure 5. The statistical results showed that the maximum, minimum, and average values of XCO_2_ concentration are 428.33, 354.54, and 405.64 ppm, respectively. The XCO_2_ concentration in the region is relatively high.

In addition, the study also conducted a correlation analysis between each variable parameter. The correlation analysis is shown in Table 2.

Through the calculation of the correlation coefficient, a certain correlation was noted between the XCO_2_ concentration and the selected modeling variables. Some variables have poor correlations, which may be attributed to the low spatial resolution of the data themselves. Data authenticity cannot be guaranteed when resampling to a finer spatial resolution. In addition, the correlation between temperature and NDVI is high, because the vegetation growth process is closely related to temperature [41]. The correlation between temperature and boundary layer height is high, mainly because temperature affects the stability of atmospheric molecules, resulting in certain changes in the boundary layer height.

### 3.2. Model Accuracy

**Figure 6:**
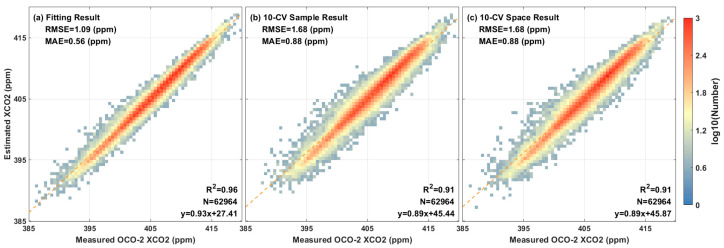
Scatter density plot of (**a**) direct fitting, (**b**) sample-based cross-validation, and (**c**) spatial cross-validation.

By establishing random forest model for the XCO_2_ reconstruction by integrating multisource remote sensing data, the model accuracy statistics were computed, including the direct fitting results of the training model, the cross-validation results based on samples, and the spatial cross-validation results based on spatial locations (Figure 6). The longitude and latitude information of each group of data were recorded. During the spatial cross-validation, all matched data were randomly divided into ten equal parts according to longitude and latitude.

The direct fitting results obtained are R^2^ = 0.96, RMSE = 1.09 ppm, and MAE = 0.56 ppm; the 10-CV results based on samples are R^2^ = 0.91, RMSE = 1.68 ppm, and MAE = 0.88 ppm; and the 10-CV results based on spatial location are R^2^ = 0.91, RMSE = 1.68 ppm, and MAE = 0.88 ppm. The validation results show that the estimation results of the model in this study are relatively close to the XCO_2_ concentration monitored by the satellite. Simultaneously, according to the results of direct fitting and 10-CV based on samples, their R^2^ values are relatively close (0.96 vs. 0.91), which can be used to judge that the model does not have a serious overfitting phenomenon. In addition, according to the 10-CV results based on spatial location (R^2^ = 0.91), it can be found that the estimation ability of the model at different positions is also outstanding. Therefore, it can be used to estimate the XCO_2_ concentration in this region.

In addition, to conduct a more detailed analysis of the accuracy of the model, the current study computed the seasonal accuracy of the model for a total of 21 seasons from 1 January 2015 to 31 December 2019. The statistical results of model accuracy by season are shown in Table 3.

Due to the influence of cloud cover and aerosols, the number of effective XCO_2_ concentration obtained in each season is different. The performance of the model in spring is poor. The mean R^2^ of the direct fitting results in the 5 years is 0.84, and the mean value of the 10-CV results is 0.64. In the 4 years from 2016 to 2019, the model accuracy in spring is the lowest. The R^2^ values of the direct fitting results are 0.81, 0.82, 0.85, and 0.81, respectively, and the R^2^ of the 10-CV results of the sample are 0.57, 0.59, 0.65, and 0.60, respectively. The performance of the model is similar in summer, autumn, and winter. The mean R^2^ values of the direct fitting results of the model in summer, autumn, and winter in the 5 years from 2015 to 2019 are 0.88, 0.90, and 0.90, respectively, and the mean values of the sample 10-CV results are 0.73, 0.77, and 0.77, respectively. The statistical results of model accuracy by season will decline to a certain extent because the model is guaranteed to be globally optimal. In addition, the MAE of the 10-CV results of the model is within 1.5 ppm for the period between the winter of 2014 and the autumn of 2019, and the average value of MAE is 0.89 ppm. It can be seen that this model can estimate regional XCO_2_ concentrations with high performance.

### 3.3. Seasonal Maps

**Figure 7:**
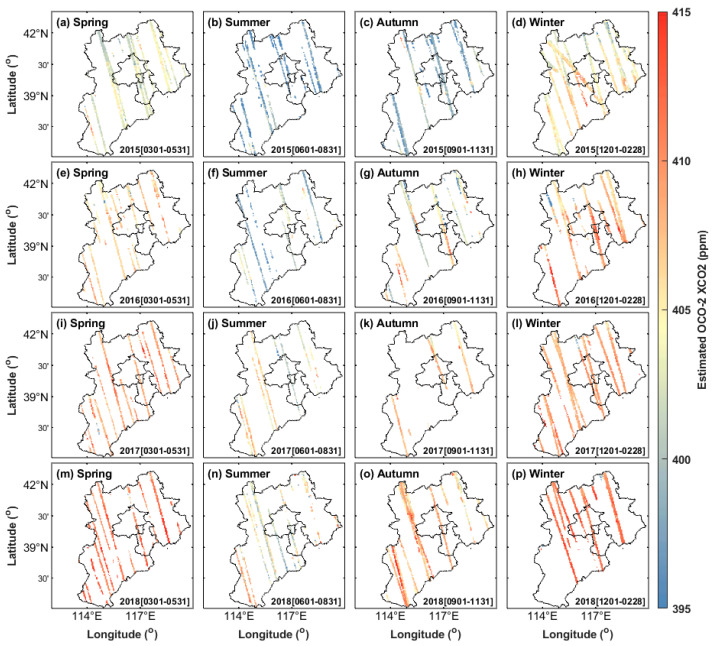
Seasonal means of OCO-2 XCO_2_ L2 Lite_FP data during 20150301–20190228, all resampled to 0.05° × 0.05° spatial resolution: (**a**–**d**) 2015, (**e**–**h**) 2016, (**i**–**l**) 2017, and (**m**–**p**) 2018 in spring, summer, autumn, and winter, respectively.

To better reflect the overall change in XCO_2_ concentration in the Beijing–Tianjin–Hebei region, the proposed model was used to estimate and map the XCO_2_ concentration in the whole region from 1 January 2015 to 31 December 2019. First of all, this study used the original OCO-2 satellite observation data to map the seasonal mean values of XCO_2_ concentration in Beijing, Tianjin, and Hebei. Since the winter data in 2019 are only in December, only the seasonal mean value results of OCO-2 XCO_2_ concentration in spring, summer, autumn, and winter from 2015 to 2018 are plotted (Figure 7).

Figure 7 shows that the coverage of the original OCO-2 XCO_2_ data in the Beijing–Tianjin–Hebei region is very low, and effective XCO_2_ monitoring cannot be conducted in many regions. Simultaneously, the return period of the OCO-2 satellite is 16 days, and XCO_2_ concentration data are only obtained once in 16 days. Due to the low coverage degree of original satellite observations, it is difficult to reflect the situation of the carbon source and carbon sink in the region. The XCO_2_ satellite observation results, as shown in Figure 7, show that the XCO_2_ concentration in the region has seasonal periodic change characteristics, and it is high in winter and spring and low in summer and autumn.

**Figure 8:**
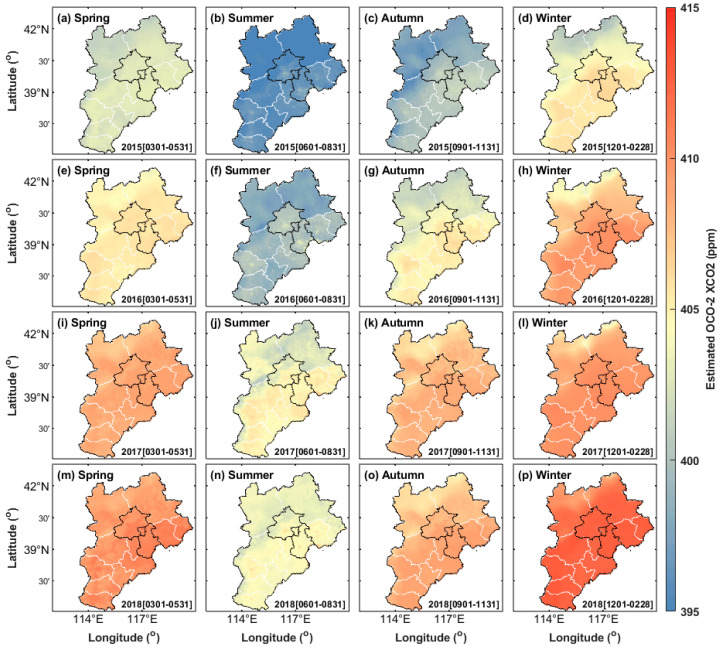
Seasonal XCO_2_ in the Beijing–Tianjin–Hebei region estimated by the random forest model from March 1, 2015 to February 28, 2019: (**a**–**d**) 2015, (**e**–**h**) 2016, (**i**–**l**) 2017, and (**m**–**p**) 2018 in spring, summer, autumn, and winter, respectively.

Secondly, the proposed model and multisource remote sensing satellite data were used to estimate the XCO_2_ concentration in the region and map the seasonal mean of the XCO_2_ concentration from 2015 to 2018 (Figure 8).

Figure 8 shows that compared with the XCO_2_ data directly observed by the OCO-2 satellite, the XCO_2_ reconstruction model established in this study can estimate the regional XCO_2_ concentration with the complete spatial distribution and can conduct more accurate studies on the regional carbon source and sink. In addition, the time resolution of the XCO_2_ concentration obtained in this study is 1 day, which can carry out more precise detection in the time dimension and effectively monitor the short-term anomaly of CO_2_ emissions.

Simultaneously, a quantitative analysis of the seasonal mean values of the XCO_2_ concentration monitored by the OCO-2 satellite and the XCO_2_ concentration estimated by the random forest model was conducted. Since the winter data in 2019 are only 1 month’s data, statistics were not computed here. The statistical results of other seasons are shown in Table 4.

Table 4 shows that little difference exists between the seasonal mean values of XCO_2_ concentration estimated by the random forest model and the seasonal mean values of XCO_2_ concentration observed by the OCO-2 satellite. The maximum difference in the mean value occurred in the spring of 2018, reaching 1.42 ppm, and the minimum difference in mean value occurred in the autumn of 2016, with a difference of only 0.03 ppm. Simultaneously, the seasonal median values of the two groups of data were calculated. Moreover, Table 4 shows that the maximum value of the median difference also appeared in the spring of 2018, reaching 1.23 ppm, and the minimum value of the difference appeared in the spring of 2017, with a difference of only 0.03 ppm. The statistical results also show that the XCO_2_ concentration was higher in spring and winter every year, followed by autumn, and smallest in summer, with periodic changes, and this is completely compatible with the findings of Yingying et al. and Bie et al. [6,42]. In this area, a dense population, high anthropogenic CO_2_ emissions, and major grain-producing areas in North China exist. However, severe seasonal changes in crops [43] and human activities make the regional seasonal change range in this area reach 9 ppm.

### 3.4. Long-Term Pattern of XCO_2_ Concentration

**Figure 9:**
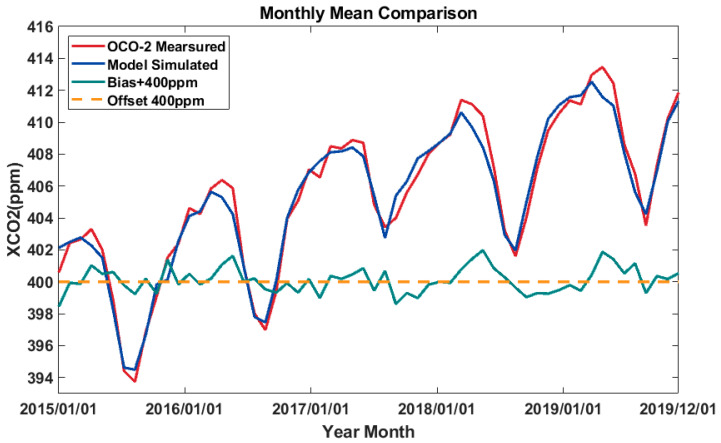
Comparison between monthly XCO_2_ concentrations from the OCO-2 satellite (red line) and the random forest model (blue line), as well as the deviation value (green line), where the deviation value increased by 400 ppm (yellow dotted line).

To make a more detail comparison between the XCO_2_ concentration monitored by the OCO-2 satellite and the XCO_2_ concentration estimated by the random forest model, the monthly mean values of the XCO_2_ concentration were also determined in this study. The results are shown in Figure 9.

Figure 9 shows that the monthly mean values of the XCO_2_ concentration estimated by this model are in good agreement with the XCO_2_ concentrations observed by the OCO-2 satellite. A large concentration deviation of the two groups of data generally occurs in the peak area of each cycle (i.e., around April and May of each year). By comparing the monthly mean values of the two groups of data, it can be found that the XCO_2_ concentration estimated by this model is consistent with the XCO_2_ concentration observed by the OCO-2 satellite. All monthly deviations are around 2 ppm, and the average absolute value of all deviations is 0.53 ppm. Simultaneously, the monthly mean concentration changes observed by the satellite and estimated by the model were compared in this study. The results are shown in Table 5.

Table 5 shows that the minimum monthly mean values of the XCO_2_ concentration observed by satellite and estimated by the model in the region appeared in August 2015, with concentrations of 393.73 and 394.10 ppm, respectively. The maximum monthly mean value of the XCO_2_ concentration observed by the satellite appeared in April 2019, with a concentration of 413.46 ppm. The maximum monthly mean value of XCO_2_ concentration estimated by the model appeared in March 2019, with a concentration of 413.00 ppm. The minimum difference in the monthly mean values of the XCO_2_ concentration observed by the satellite and estimated by the model was about 0.00 ppm, which occurred in October 2016, and the maximum difference occurred in November 2015, which was 1.67 ppm.

### 3.5. Spatial Distribution of Monthly XCO_2_ Concentration

**Figure 10:**
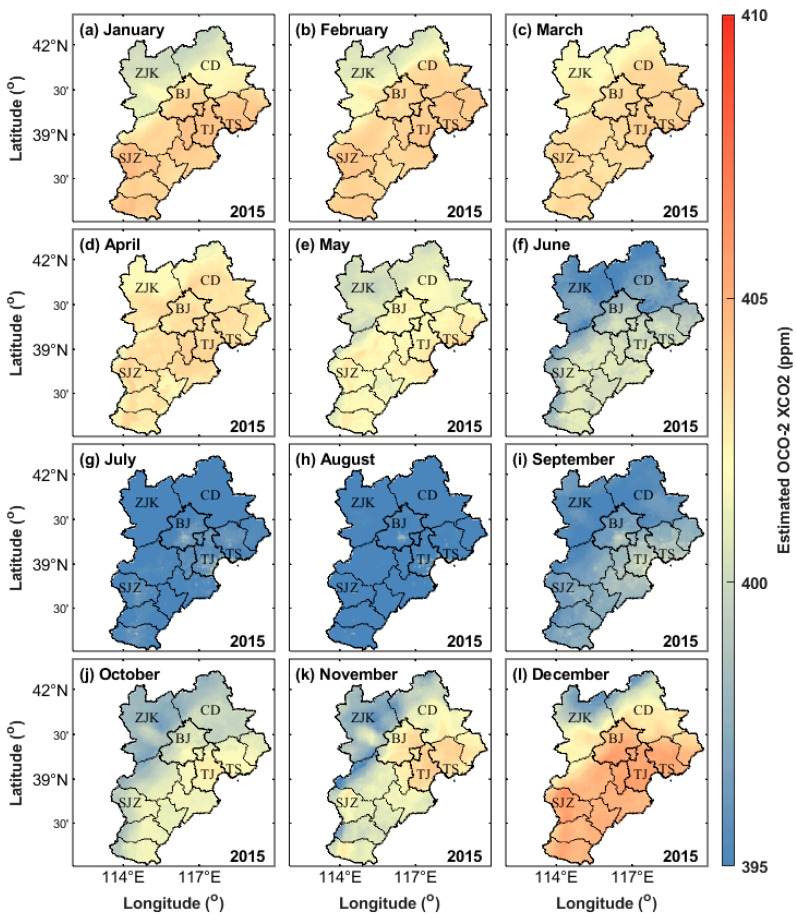
Monthly average XCO_2_ concentrations in the Beijing–Tianjin–Hebei region from January 2015 to December 2015. ZJK, CD, BJ, TJ, TS, and SJZ represent Zhangjiakou, Chengde, Beijing, Tianjin, Tangshan, and Shijiazhuang, respectively. (**a**–**l**) represent January to December respectively.

**Figure 11:**
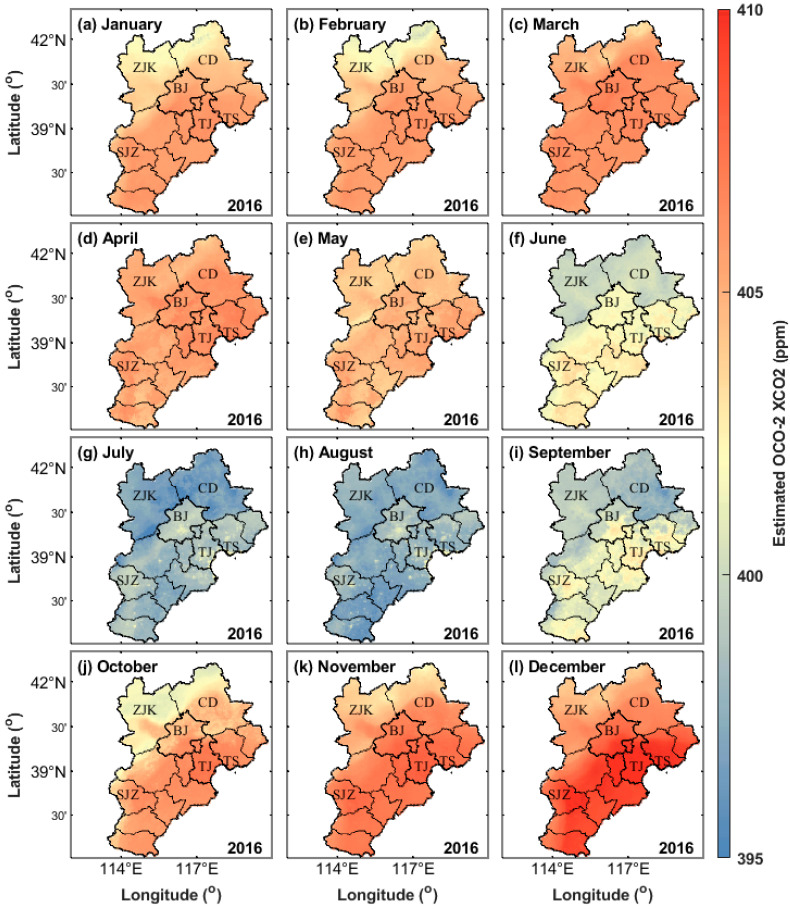
Monthly average XCO_2_ concentrations in the Beijing–Tianjin–Hebei region from January 2016 to December 2016. ZJK, CD, BJ, TJ, TS, and SJZ represent Zhangjiakou, Chengde, Beijing, Tianjin, Tangshan, and Shijiazhuang, respectively. (**a**–**l**) represent January to December respectively.

To show the temporal and spatial changes in XCO_2_ concentration in this study, the monthly maps of the XCO_2_ concentration in 2015 and 2016 are drawn (Figure 10 and Figure 11).

Figure 10 and Figure 11 show that the XCO_2_ concentration in the Beijing–Tianjin–Hebei region shows fluctuations. Simultaneously, it has a rhythm: the XCO_2_ concentration is higher in spring and winter, followed by autumn, and the lowest in summer, which has a rhythm of seasonal change.

According to the monthly change in net primary productivity in the Beijing–Tianjin–Hebei region, Quanhong [44] pointed out that the vegetation in this region recovers in spring and enters the growth season. After summer, the water and heat conditions are suitable, the vegetation grows vigorously, the ecosystem productivity is the best, and the carbon fixation capacity is the strongest. In autumn, due to the maturity of agricultural crops, the ecological productivity of the whole region gradually decreases.

The high XCO_2_ concentration from March to May may be caused by the CO_2_, CH_4_, and other gases released by the decaying litter of forest vegetation. The low XCO_2_ concentration from July to September is mainly caused by a large amount of CO_2_ absorbed by forest vegetation during the growth process. The CO_2_ release from forest vegetation is greater than the absorption from March to June every year, while the CO_2_ absorption of forest vegetation from July to October is greater than the release. Therefore, in the process of the carbon cycle, the carbon source is the main feature in spring, and the carbon sink is the main feature in summer and autumn. In spring, plants begin to grow and absorb CO_2_ in the atmosphere but are offset by CO_2_ released into the atmosphere by plant decay. These plants do not completely decay between the colder late autumn and winter due to the low activity of humus organisms.

In addition, compared with the banded XCO_2_ concentration observed by the OCO-2 satellite, some carbon source and sink regions can be effectively reflected by the seamless XCO_2_ concentration monitored by the model of the Beijing–Tianjin–Hebei region. Figure 10 and Figure 11 show that some areas in Beijing, Tianjin, Tangshan, and Shijiazhuang are carbon source areas, and their monthly average XCO_2_ concentrations are significantly higher than those of the surrounding areas. The main reason may be that the above cities have large populations and large anthropogenic emissions. In some areas, such as Zhangjiakou and Chengde, the monthly XCO_2_ concentration is significantly lower than that of the surrounding areas. The main reason may be that the above two cities are underdeveloped, have a small residential population, and have relatively low industrial CO_2_ emissions.

## 4. Discussion

Many models have been established to estimate regional CO_2_ concentrations to better reveal the change in atmospheric CO_2_ concentration. Guo modeled the spatial distribution of XCO_2_ in five continents, considering temperature and vegetable cover [45]. However, the highest R^2^ was 0.75 in Eurasia, which is not sufficient to meet the requirements of high-performance CO_2_ concentration analysis. With the development of artificial intelligence, machine learning models have been used in XCO_2_ concentration monitoring. Saibi et al. [25] modeled the spatial distribution of XCO_2_ to assess the spatial distribution of CO_2_ concentration during the growing seasons in Iran, considering meteorological factors and natural carbon sink factors. However, the highest and lowest R^2^ values were 0.77 and 0.38 for April and September, respectively.

To better estimate CO_2_ concentration, more influencing factors and model performance need to be considered. The random forest model, based on the consideration of time series factors, meteorological factors, anthropogenic emission factors, natural carbon sink factors, and other factors affecting atmospheric CO_2_ concentration, can achieve higher R^2^ (0.96) and 10-CV R^2^ (0.91) than other models (0.77 and 0.75). This high-precision model can be used to estimate the XCO_2_ concentration, which can better reflect the changing trend and spatial distribution of atmospheric CO_2_ concentration in the study area.

In addition, the observation data of the OCO-2 satellite were mainly used to model and estimate the CO_2_ concentration in the Beijing–Tianjin–Hebei region in the study. However, due to the insufficient spatial resolution of the OCO-2 satellite, the spatial resolution of regional CO_2_ concentration obtained in this study is not sufficient to support the carbon emission monitoring of large-scale power plants and coal-fired plants. Thanks to the continuous development of remote sensing satellites, CO_2_ satellite monitoring data with higher spatial resolution and higher accuracy are being continuously retrieved. In the next work, more CO_2_ satellites, such as GF-5 and OCO-3 satellites, will be combined to retrieve higher-quality CO_2_ data to achieve the monitoring of plant carbon emissions.

## 5. Conclusions

CO_2_ is the most abundant greenhouse gas in the atmosphere, and its rising concentration has caused various climate changes and natural disasters, which have attracted extensive attention. Since the 1970s, the means of monitoring atmospheric CO_2_ have been continuously developed and updated. From station monitoring to satellite observation, from surface concentration to column concentration, the accurate estimation of atmospheric CO_2_ concentration and the accurate identification of regional and even global carbon source and sink locations require high-precision, high-spatial–temporal-resolution, and high-coverage atmospheric CO_2_ concentration monitoring data. In this study, multiple sources of atmospheric CO_2_ were considered, multisource remote sensing data were fused, and the random forest algorithm was used to build a high-coverage reconstruction model of XCO_2_ concentration, and temporal and spatial differences in the XCO_2_ concentration data set in the Beijing–Tianjin–Hebei region obtained from the model were analyzed. The main achievements are as follows:

1. Aiming at the problems of the low spatial coverage and insufficient temporal resolution of the XCO_2_ concentration observation data obtained by the OCO-2 monitoring satellite, this study developed a high-coverage reconstruction model for XCO_2_ concentration by integrating multisource remote sensing data. Simultaneously, the accuracy of the model was evaluated. The direct fitting results are R^2^ = 0.96, RMSE = 1.09 ppm, and MAE = 0.56 ppm; the 10-CV results based on samples are R^2^ = 0.91, RMSE = 1.68 ppm, and MAE = 0.88 ppm; and the 10-CV results based on spatial location are R^2^ = 0.91, RMSE = 1.68 ppm, and MAE = 0.88 ppm. The developed model has the potential to play an important role in the monitoring of atmospheric CO_2_ concentration.
2. Using the developed model, the high-coverage daily XCO_2_ concentration with a spatial resolution of 0.05° in the Beijing–Tianjin–Hebei region from 2015 to 2019 was outputted, and the monthly and seasonal means of XCO_2_ concentration were compared with those measured by the OCO-2 satellite. The study found that the XCO_2_ concentration has obvious fluctuation and rhythm. The XCO_2_ concentration is higher in spring and winter due to the decay of litter and human emissions. With the large amount of CO_2_ absorbed by green vegetation photosynthesis, the XCO_2_ concentration in summer is lower. In addition, in terms of the spatial XCO_2_ distribution concentration, some areas in Beijing, Tianjin, Tangshan, and Shijiazhuang are carbon source areas, and their monthly average XCO_2_ concentrations are significantly higher than those of the surrounding areas.

In general, this model has the potential to play a role in estimating the change in regional XCO_2_ concentration, monitoring the location of carbon sources and to help constrain city emissions on city scales.

## Figures and Tables

**Table 1:** Data sets used in this study.

Data	Variable	Unit	Time Resolution	Spatial Resolution	Source
XCO_2_	XCO_2_	ppm	1 day	2.25 km × 1.29 km	OCO-2
Lighting data	DN	Unitless	1 day	500 m	S-NPP
Carbon sink	NDVI	Unitless	16 days	500 m	MODIS
	RELH	%	1 h	0.25°	
Meteorological data	TEMP	K	1 h	0.25°	ERA5
	WS	m/s	1 h	0.25°	
	PRES	Kpa	1 h	0.25°	
	BLH	km	1 h	0.25°	

**Table 2:** Pearson correlation coefficient matrix of XCO_2_ concentration reconstruction model with matching data set variables.

	Time	XCO_2_	TEMP	RELH	PRES	uwind	vwind	BLH	NDVI	DN
**Time**	1.00	0.62	0.07	−0.10	−0.02	0.03	0.01	0.07	0.07	0.00
**X** **CO_2_**		1.00	−0.21	−0.30	0.16	0.10	0.03	−0.01	−0.26	0.08
**TEMP**			1.00	0.05	0.22	−0.39	0.29	0.42	0.65	0.08
**RELH**				1.00	0.04	−0.24	0.18	−0.49	0.13	0.00
**PRES**					1.00	−0.33	0.08	−0.10	0.11	0.37
**uwind**						1.00	−0.17	0.19	−0.30	−0.11
**vwind**							1.00	−0.26	0.16	0.01
**BLH**								1.00	0.24	−0.01
**NDVI**									1.00	0.05
**DN**										1.00

**Table 3:** Statistical results of seasonal accuracy of the model from 1 January 2015 to 31 December 2019 (21 seasons, n = 62,964 is the total number of samples, and the evaluation indicators are R^2^, MAE, and RMSE).

Season	Direct Fitting Results			10-CV Results Based on Samples			Number
	R^2^	MAE (ppm)	RMSE (ppm)	R^2^	MAE (ppm)	RMSE (ppm)	
2014 Winter	0.89	0.43	0.87	0.77	0.65	1.25	2078
2015 Spring	0.91	0.49	0.91	0.79	0.73	1.35	3603
2015 Summer	0.89	0.83	1.43	0.73	1.27	2.14	2913
2015 Autumn	0.88	0.61	1.25	0.70	0.94	1.93	3342
2015 Winter	0.92	0.53	1.09	0.81	0.82	1.66	6015
2016 Spring	0.81	0.53	0.94	0.57	0.81	1.37	2586
2016 Summer	0.87	0.87	1.61	0.71	1.30	2.37	2318
2016 Autumn	0.93	0.57	1.17	0.82	0.87	1.76	3200
2016 Winter	0.93	0.50	1.00	0.82	0.78	1.57	3703
2017 Spring	0.82	0.55	1.04	0.59	0.83	1.51	3158
2017 Summer	0.91	0.72	1.22	0.79	1.11	1.82	1700
2017 Autumn	0.89	0.61	1.23	0.72	0.93	1.85	1702
2017 Winter	0.86	0.47	1.07	0.68	0.70	1.56	4971
2018 Spring	0.85	0.45	0.82	0.65	0.68	1.22	2789
2018 Summer	0.85	0.98	1.64	0.66	1.48	2.43	2509
2018 Autumn	0.90	0.51	0.99	0.78	0.76	1.44	3894
2018 Winter	0.90	0.44	0.84	0.74	0.66	1.30	2899
2019 Spring	0.81	0.51	1.06	0.60	0.75	1.46	3147
2019 Summer	0.90	0.86	1.51	0.76	1.29	2.20	2020
2019 Autumn	0.92	0.43	0.78	0.83	0.64	1.15	3493
2019 Winter	0.92	0.51	0.94	0.82	0.76	1.38	924

**Table 4:** Statistical results of the seasonal mean values of the XCO_2_ concentration monitored by the OCO-2 satellite and the XCO_2_ concentration estimated by the random forest model.

Season	Monitored by Satellite			Estimated by Model			Bias		
	Mean	Median	Standard Deviation	Mean	Median	Standard Deviation	Mean	Median	Standard Deviation
201501	402.59	402.77	2.85	402.40	402.66	0.75	0.18	0.11	2.10
201502	395.50	395.55	4.10	395.39	395.61	0.83	0.11	−0.06	3.27
201503	398.74	398.73	3.30	398.59	399.00	1.67	0.15	−0.27	1.63
201504	404.13	404.19	3.10	403.77	404.01	1.86	0.36	0.18	1.24
201601	406.02	406.13	2.00	405.42	405.55	0.54	0.60	0.58	1.47
201602	398.50	398.41	4.44	399.19	399.29	0.92	−0.69	−0.88	3.52
201603	403.15	403.49	3.85	403.18	402.92	1.36	−0.03	0.57	2.49
201604	407.43	407.52	3.77	407.66	407.80	1.91	−0.23	−0.28	1.86
201701	408.68	408.58	2.25	408.24	408.55	0.86	0.44	0.03	1.39
201702	404.18	404.47	3.84	404.33	404.43	0.94	−0.15	0.04	2.91
201703	406.24	406.68	3.05	406.70	407.17	1.17	−0.46	−0.49	1.89
201704	408.77	408.84	2.71	408.75	409.23	1.52	0.02	−0.39	1.19
201801	411.09	410.99	2.01	409.67	409.76	0.85	1.42	1.23	1.16
201802	404.09	404.06	4.18	403.71	403.69	0.46	0.39	0.37	3.72
201803	407.22	407.35	3.00	407.53	407.86	1.13	−0.31	−0.51	1.87
201804	411.05	411.08	2.59	411.35	411.91	1.29	−0.30	−0.83	1.30
201901	412.96	412.86	2.10	411.94	411.94	0.54	1.02	0.92	1.56
201902	406.09	406.26	4.43	406.33	406.46	0.86	−0.24	−0.20	3.57
201903	409.63	409.72	2.73	409.35	409.89	1.16	0.27	−0.17	1.58

**Table 5:** The monthly mean concentration changes observed by the satellite and estimated by the model.

**Observed by the OCO-2 satellite**	Minimum	393.73	201508
	Maximum	413.46	201904
	Bias	19.73	
**Estimated by the random forest model**	Minimum	394.10	201508
	Maximum	413.00	201903
	Bias	18.94	
**Bias**	Minimum	0.00	201610
	Maximum	1.67	201511

## Data Availability

OCO-2 XCO_2_ data supporting the reported results can be found and downloaded from website (https://search.earthdata.nasa.gov, accessed on 10 February 2021).
